# Repeated local emergence of carbapenem-resistant *Acinetobacter baumannii* in a single hospital ward

**DOI:** 10.1099/mgen.0.000050

**Published:** 2016-03-02

**Authors:** Mark B. Schultz, Duy Pham Thanh, Nhu Tran Do Hoan, Ryan R. Wick, Danielle J. Ingle, Jane Hawkey, David J. Edwards, Johanna J. Kenyon, Nguyen Phu Huong Lan, James I. Campbell, Guy Thwaites, Nguyen Thi Khanh Nhu, Ruth M. Hall, Alexandre Fournier-Level, Stephen Baker, Kathryn E. Holt

**Affiliations:** ^1^​ Department of Biochemistry and Molecular Biology, Bio21 Molecular Science and Biotechnology Institute, University of Melbourne, Parkville, Victoria 3010, Australia; ^2^​ Centre for Systems Genomics, University of Melbourne, Parkville, Victoria 3010, Australia; ^3^​ The Hospital for Tropical Diseases, Wellcome Trust Major Overseas Programme, Oxford University Clinical Research Unit, Ho Chi Minh City, Vietnam; ^4^​ School of Molecular Bioscience, University of Sydney, New South Wales, Australia; ^5^​ School of Biomedical Science, Queensland University of Technology, Queensland, Australia; ^6^​ Centre for Tropical Medicine, Nuffield Department of Medicine, Oxford University, London, UK; ^7^​ Department of Genetics, University of Melbourne, Parkville, Victoria 3010, Australia

**Keywords:** capsule switching, hospital acquired infection, imipenem resistance, local evolution, molecular epidemiology, phylogenomic analysis

## Abstract

We recently reported a dramatic increase in the prevalence of carbapenem-resistant *Acinetobacter baumannii* infections in the intensive care unit (ICU) of a Vietnamese hospital. This upsurge was associated with a specific *oxa23*-positive clone that was identified by multilocus VNTR analysis. Here, we used whole-genome sequence analysis to dissect the emergence of carbapenem-resistant *A. baumannii* causing ventilator-associated pneumonia (VAP) in the ICU during 2009–2012. To provide historical context and distinguish microevolution from strain introduction, we compared these genomes with those of *A. baumannii* asymptomatic carriage and VAP isolates from this same ICU collected during 2003–2007. We identified diverse lineages co-circulating over many years. Carbapenem resistance was associated with the presence of *oxa23*, *oxa40, oxa58* and *ndm1* genes in multiple lineages. The majority of resistant isolates were *oxa23*-positive global clone GC2; fine-scale phylogenomic analysis revealed five distinct GC2 sublineages within the ICU that had evolved locally via independent chromosomal insertions of *oxa23* transposons. The increase in infections caused by carbapenem-resistant *A. baumannii* was associated with transposon-mediated transmission of a carbapenemase gene, rather than clonal expansion or spread of a carbapenemase-harbouring plasmid. Additionally, we found evidence of homologous recombination creating diversity within the local GC2 population, including several events resulting in replacement of the capsule locus. We identified likely donors of the imported capsule locus sequences amongst the *A. baumannii* isolated on the same ward, suggesting that diversification was largely facilitated via reassortment and sharing of genetic material within the localized *A. baumannii* population.

## Data Summary

1. Supplementary data (Table S1, Figs S1–S6, File S1, File S2 and Note S1) have been deposited in FigShare: 10.6084/m9.figshare.2064357
2. Illumina paired-end short-read sequences reported in this paper have been deposited in the European Nucleotide Archive under project ERP001080. Individual isolate accession numbers are provided in Table S1.
3. Novel annotated sequences have been submitted to GenBank as plasmid p255n_1 (accession number KT852971), plasmid pBAL_204 (accession number KT946773), transposon Tn*2008VAR* (accession number KT852972), capsule loci KL32 (accession number KT359615), KL49 (accession number KT359616) and KL58 (accession number KT359617). The novel insertion sequence was submitted to ISfinder as IS*Aba33*. Schematic representations of novel annotated sequences have been deposited in FigShare: 10.6084/m9.figshare.2064357
4. Tree files (Bayesian and maximum-likelihood), metadata (CSV) and Gubbins recombinant blocks (GFF3) files for display in Phandango (previously known as JScandy; http://jameshadfield.github.io/phandango/) are deposited in FigShare: 10.6084/m9.figshare.2064357

## Impact Statement

Infections caused by antibiotic-resistant bacteria pose a heavy burden on human health globally. Where antibiotics are in sustained usage, bacteria have been shown to rapidly evolve to become drug resistant. The problem of antibiotic-resistant bacterial infections in intensive care units (ICUs) is becoming increasingly common and understanding the means by which bacteria develop antibiotic resistance in the ICU may help to prevent or slow this process in the future, extending the life of new antibiotics. *Acinetobacter baumannii* is one of the most problematic bacterial species that cause antibiotic-resistant infections in ICU patients. In this study, we used genome sequencing to retrace the evolution of resistance to an important class of antibiotics (carbapenems) amongst *A. baumannii* bacteria circulating in an ICU ward in Vietnam. We found that after carbapenems were introduced as a common treatment for infections in the ICU in 2008, several different strains of *A. baumannii* that were susceptible to carbapenems in earlier years became resistant by acquiring new genes, which encoded carbapenemase enzymes that break down carbapenem-based drugs. These newly resistant strains became very common on the ward in later years, so that most infections could no longer be effectively treated using carbapenems. In finding strong evidence for the repeated local emergence of drug resistance in response to local usage of the drug, our findings provide a predictive framework for healthcare practitioners dealing with these types of infection.

## Introduction

Over the past three decades, *Acinetobacter baumannii* has become increasingly implicated in outbreaks of multiply antibiotic-resistant hospital-acquired infections worldwide (Antunes *et al.*, 2014; Bouvet & Grimont, 1987; Perez *et al.*, 2007, 2011). The organism is now universally recognized as one of the six most problematic hospital-adapted antimicrobial-resistant bacterial pathogens, contributing the ‘A’ in the ‘ESKAPE’ pathogens (Rice, 2008). The majority of hospital-acquired *A. baumannii* infections are caused by two globally disseminated clones, known as global clones GC1 and GC2. In intensive care units (ICUs), ventilator-associated pneumonia (VAP) is the second most common hospital-acquired infection (Kalanuria *et al.*, 2014), affecting up to a quarter of mechanically ventilated patients (Chastre & Fagon, 2002), and has an attributable mortality risk of ∼13 % (Melsen *et al.*, 2013). VAP caused by antimicrobial-resistant *A. baumannii* is becoming increasingly common in industrialized countries. Antimicrobial-resistant *A. baumannii* have been isolated in up to 45 % of VAP cases in the ICU at the Hospital for Tropical Diseases (HTD), Ho Chi Minh City, Vietnam (Nhu *et al.*, 2014).

*A. baumannii* are intrinsically resistant to chloramphenicol and florfenicol, and resistance to all other first-line antimicrobials (sulphonamides, tetracycline and ampicillin) has become largely ubiquitous amongst hospital-acquired *A. baumannii* since the 1980s (Bergogne-Bérézin & Towner, 1996). Resistance to cephalosporins and fluoroquinolones emerged in the 1990s, leaving carbapenems (particularly imipenem) as the mainstay for empirical treatment (Bergogne-Bérézin & Towner, 1996). Imipenem first became available for the treatment of microbial infections in 1985 (Papp-Wallace *et al.*, 2011). Coincidentally, imipenem-resistant *A. baumannii* were first isolated in 1985 from a hospital in which imipenem had never been used (Paton *et al.*, 1993), with resistance later found to be conferred by the *oxa23* gene (Donald *et al.*, 2000). Thus, the mechanisms of resistance to imipenem already existed in the *A. baumannii* population well before the first use of imipenem (Paton *et al.*, 1993; Poirel *et al.*, 2008). Over the last decade, imipenem-resistant *A. baumannii* infections have been observed more widely, prompting the use of colistin for antimicrobial therapy (Garonzik *et al.*, 2011). Although colistin is effective against most carbapenem-resistant *A. baumannii*, it is a last-resort treatment due to toxicity (e.g. Evans *et al.*, 2013; Ordooei Javan *et al.*, 2015; Spapen *et al.*, 2011) and resistance to colistin in *A. baumannii* is also emerging (Cai *et al.*, 2012; Qureshi *et al.*, 2015). Being naturally competent (Wilharm *et al.*, 2013) and able to form biofilms (Madsen *et al.*, 2012; McQueary *et al.*, 2012; Tomaras *et al.*, 2003), homologous recombination is common in *A. baumannii* populations; however, few genomic studies have investigated this in detail.

We recently investigated the aetiology of VAP in the ICU at the HTD (Nhu *et al.*, 2014). *Acinetobacter* was the dominant genus implicated in VAP and we documented an increase in its isolation from 28 % of VAP during 2000–2007 to 46 % in 2010 (Nhu *et al.*, 2014). Approximately 15 % of VAP-associated *Acinetobacter* were resistant to imipenem during 2002–2007. Imipenem was approved for empirical treatment of VAP in this ICU in 2008, following which the frequency of imipenem resistance increased to 46 % of *A. baumannii* VAP isolates in 2009 and 86 % in 2010 (Nhu *et al.*, 2014). Multilocus VNTR analysis (MLVA) and PCR profiling of carbapenemase genes indicated that this increase was largely attributable to *A. baumannii* sharing the same MLVA profile (MLVA6) and carrying the *oxa23* gene (Nhu *et al.*, 2014). Thus, we hypothesized that the dominant VAP-associated *A. baumannii* subtype constituted a single clone that had been introduced and then maintained on the ICU ward, likely due to sustained imipenem usage. However, MLVA data could not resolve ancestor–descendent relationships among the samples nor identify whether GC1 or GC2 were present.

Here, we used whole-genome sequencing to study the population of imipenem-resistant *A. baumannii* isolated from VAP on the HTD ICU during 2008–2012 and to investigate their relationship to imipenem-susceptible *A. baumannii* isolated from VAP or asymptomatic carriage on the same ICU ward in 2003–2007.

## Methods

### Bacterial isolates and DNA sequencing

To examine the relationships between *A. baumannii* carbapenem-resistant and -susceptible isolates, we sequenced the genomes of isolates collected during two different studies (from two different time periods) in the same ICU ward at the HTD. This ICU is largely devoted to the treatment of tetanus patients (Nhu *et al.*, 2014). The first study aimed to characterize asymptomatic colonization (carriage) of patients by potentially pathogenic commensal bacteria (including *Acinetobacter* spp., *Klebsiella pneumoniae*, *Pseudomonas aeruginosa* and *Staphylococcus aureus*) during 2003–2007 (Schultsz *et al.*, 2013). The following swabs were collected from patients entering the ward within 48 h of admission: nasal swab (‘n’ or ‘N’), axilla swab (‘ax’), anal swab (‘an’) and skin swabs at the cannula tip point of entry (‘c’). These swabs yielded 70 *A. baumannii* asymptomatic carriage isolates available for sequencing. A total of 12 *A. baumannii* VAP isolates from this period were identified in a review of laboratory inventory, so these were also sequenced for comparison. The second study (described in Nhu *et al.*, 2014) aimed to understand the bacterial aetiology of emerging drug-resistant VAP on the ward and reviewed bacterial isolates cultured from specimens from intubated patients via bronchoalveolar lavage (BAL) or tracheal washes (UV). From this latter study, a total of 77 *A. baumannii* isolated during 2008–2012 were available for sequencing. These isolates were all considered to be causative agents of VAP; no asymptomatic carriage isolates were collected during this time period. The list of all 159 *A. baumannii* isolates that were retrospectively combined for the present study is provided in Table S1.

Genomic DNA was extracted using a Wizard Genomic Extraction kit (Promega) and subjected to 96-plex barcoded 100 bp paired-end sequencing via Illumina HiSeq 2500, as described previously (Holt *et al.*, 2012). Twelve of the infection isolates failed sequencing, leaving a total of 147 genomes for analysis. Sequence reads are available in the European Nucleotide Archive under project ERP001080. Individual accession numbers are provided in Table S1.

### Antimicrobial susceptibility testing

Antimicrobial susceptibilities were determined at the time of isolation by the modified Kirby–Bauer disk diffusion method, as recommended by the Clinical and Laboratory Standards Institute (CLSI, 2011): piperacillin/tazobactam (100/10 μg), imipenem (10 μg), amikacin (30 μg), ceftazidime (30 μg), ceftriaxone (30 μg), ciprofloxacin (5 μg), gentamicin (30 μg) and colistin (10 μg). Mueller–Hinton agar and antimicrobial discs were purchased from Unipath. For colistin, imipenem, ceftazidime and ceftriaxone, MICs were determined by E-test based on the manufacturer's recommendations (AB Biodisk). The results were interpreted as resistant or sensitive according to current Clinical and Laboratory Standards Institute guidelines. *Escherichia coli* ATCC 25922 was used as the control for these assays.

### SNP identification and phylogenomic analysis

Whole-genome sequences were inferred by mapping Illumina reads against the 3 940 614 bp reference genome of *A. baumannii* GC2 strain 1656-2 (GenBank accession number CP001921.1) using the RedDog mapping pipeline (https://github.com/katholt/RedDog). Briefly, RedDog uses Bowtie version 2.2.3 (Langmead & Salzberg, 2012) to map reads to the reference sequence, then high-quality SNPs with Phred quality score ≥ 30 are extracted from the resulting alignments using SAMtools version 0.1.19. SNPs were filtered to exclude those with five or more reads mapped or with >2.5 times the mean read depth (representing putative repeated sequences), or with heterozygous (ambiguous) allele calls. For each SNP that passed these criteria in any one isolate, consensus allele calls for the SNP locus were extracted from all genomes (ambiguous base calls and bases with Phred quality < 20 were treated as unknown alleles and represented with a gap character). SNPs with confident homozygous allele calls (Phred quality ≥ 20 calculated by SAMtools consensus) in >90 % of the *A. baumannii* genomes (representing a ‘soft’ core genome of common *A. baumannii* sequences) were concatenated to produce an alignment of 217 290 variant sites.

Maximum-likelihood (ML) analysis was performed using the GTR model with a gamma distribution of rate heterogeneity (GTR+G) using RAxML version 8.1.16 (Stamatakis, 2014). We modelled rate heterogeneity due to the high level of recombination known to occur in *A. baumannii* (Madsen *et al.*, 2012). We did not apply an ascertainment bias correction because the variable sites under analysis were those obtained from whole-genome sequencing and not simply from typing of known variable sites. ML analyses were performed 10 times with 100 bootstrap replicates per run. Likelihood scores were compared between runs to confirm that all runs had converged on a similar likelihood; we selected the single tree with the highest likelihood as the best tree.

From the SNP alignment of 147 isolates, we extracted a SNP alignment for the 66 GC2 isolates including only the 13 950 SNPs that varied amongst these GC2 genomes. RAxML analyses were repeated 10 times with 100 bootstrap replicates per run. For these analyses we used empirical base frequencies and a GTR best-fit model of nucleotide substitution. The substitution model was estimated under the Akaike information criterion (AIC; Akaike, 1974) using MrModeltest version 2.3 (https://github.com/nylander/MrModeltest2) in paup* (http://paup.csit.fsu.edu/). Temporal signal (‘clock-likeness’) captured in the ML trees was assessed using Path-O-Gen version 1.4 (Drummond *et al.*, 2012), which performs linear regression of root-to-tip ML branch distances (*y*) on year of isolation (*x*). Tree topologies were rooted prior to temporal analysis using an outgroup from clonal complex CC10.

### Visualization of recombination

In order to visualize clusters of SNPs that were likely imported into the *A. baumannii* genomes via recombination events, we generated plots of SNP density distributions along the GC2 strain 1656-2 reference genome using r (version 3.2.1). Taking the first isolate in the alignment as a baseline sequence, we calculated the number of SNPs between the baseline sequence and each other isolate, in 1 kbp windows. The resulting values, which represent density of SNPs per 1 kbp along the reference genome, were plotted as a heatmap, ordered against the ML tree and output to a PNG image file. This process was applied iteratively, taking each isolate in turn as the baseline genome. The resulting sequence of PNG files was combined into an image-sequence movie file (File S1).

Additionally, we inferred split networks to assess tree-likeness of the phylogenetic structure in the SNP alignments using SplitsTree4 (Huson & Bryant, 2006) with default settings (uncorrected-*p* distance, cyclic splits computed using NeighbourNet and ordinary least-squares variance).

### Recovering a recombination-free phylogenetic tree for GC2

We made a number of attempts to detect recombinant sites within the GC2 population. These analyses were performed on a pseudo-whole-genome alignment for GC2 in which each isolate genome was reconstructed by copying the reference sequence and replacing the SNP sites with alleles from the isolate in question. Note that after the recombination detection analysis, invariant sites were removed and the alignment was reduced to those common genomic sites that were present in ≥ 90 % of the isolates under study.

Recombinant sites were identified and removed using: (i) ClonalFrameML (Didelot & Wilson, 2015) with default settings and 100 bootstrap replicates, and (ii) v1.1.2 (Croucher *et al.*, 2015) for 20 iterations with default settings. The resultant recombination-free alignments of variant sites were inspected using SplitsTree4 to build split networks. In addition, as we expected a strong temporal signal within the GC2 clone similar to that observed within other Gram-negative clones, such as *Shigella sonnei* (Holt *et al.*, 2012), *Vibrio cholerae* (Mutreja *et al.*, 2011) or *Salmonella enterica* Typhimurium (Okoro *et al.*, 2012), we assessed the strength of temporal signal before and after the various recombination detection procedures by inferring ML trees from the variable sites in the alignments using RAxML and then analysing these in Path-O-Gen version 1.4 (Drummond *et al.*, 2012). As the tree obtained after Gubbins analysis recovered the strongest temporal signal, we utilized the Gubbins-filtered alignment for further analysis.

We performed time-calibrated Bayesian phylogenetic inference on the post-Gubbins SNP alignment (457 sites) using beast version 1.8.0 with the AIC-selected GTR+G best-fit substitution model, empirical base frequencies, a strict molecular clock and a coalescent (constant size) tree prior. beast Markov Chain Monte Carlo (MCMC) analyses were repeated eight times starting each run from a random seed and a random tree. MCMC analyses were continued for 10 × 10^6^ iterations, sampling every 1000 iterations. After removing a burn-in of 100 samples (i.e. 1 × 10^6^ MCMC iterations) from each file, logs were combined using LogCombiner version 1.8 to give 72 000 post-burn-in samples (i.e. 72 × 10^6^ MCMC iterations). Effective sample sizes (ESSs) for all parameters of interest were >>1000 as determined by analysis with Tracer version 1.6. Burn-in was 10 % of the chain length. Post-burn-in trees were combined using LogCombiner and summarized using TreeAnnotator version 1.8. The common ancestor summary tree was then recovered from the set of trees (*n* = 72 000 trees) in the posterior sample so that every clade in the summary tree was assigned an age using the mean of the clade age in all posterior trees (Heled & Bouckaert, 2013).

### MLST analysis and detection of acquired resistance genes from sequence reads

srst2 (Inouye *et al.*, 2014) was used with default settings to identify MLSTs and acquired resistance genes directly from reads. For MLST analysis, we used both the Institut Pasteur and University of Oxford MLST databases (http://pubmlst.org/abaumannii/; downloaded 3 June 2015). All sequence types reported in the text are based on the Pasteur scheme; however, those inferred using the Oxford scheme are also provided for comparison in Table S1. For acquired resistance genes we used the srst2-formatted version of the arg-annot database (Gupta *et al.*, 2014), distributed with srst2 (Inouye *et al.*, 2014).

### Genome assembly

Each 100 bp paired-end read set was assembled *de novo* using SPAdes version 3.6.1 (Bankevich *et al.*, 2012), with *k*-mer sizes 21, 33, 55, 77 and 91, error correction with BayesHammer (Langmead & Salzberg, 2012), and mismatch correction.

### Genetic context and copy number of carbapenemase genes

The genetic contexts of carbapenemase genes (*oxa23*, *oxa40*, *oxa58* and *ndm1*) were explored in detail via manual inspection of the SPAdes assembly graphs using Bandage version 0.7.0 (Wick *et al.*, 2015). All isolates carrying the *oxa23* gene were confirmed to carry the gene either within Tn*2006* (Corvec *et al.*, 2007) or a variant of Tn*2008* (Adams-Haduch *et al.*, 2008). These transposons are mobilized by IS*Aba1* (GenBank accession number AY758396) and it was not possible to determine, using the Illumina short reads, which of the multiple insertions of IS*Aba1* in each genome were associated with the transposon and which represented other IS*Aba1* insertions. However, we were able to investigate the IS*Aba1* insertion sites in detail using ISMapper (Hawkey *et al.*, 2015) to identify all insertion sites for IS*Aba1* relative to the reference chromosome sequence (GC2 strain 1656-2; GenBank accession number CP001921.1) and using Bandage to investigate whether IS*Aba1* was present within additional locations, including the Aci6 plasmid (pA85-3 from a GC1 strain; GenBank accession number KJ493819.1). As *oxa23* was only found in the chromosome, copy number of *oxa23* was inferred from the distribution of ratios of *oxa23* mapping depths to respective mean chromosome mapping depths. In the cases of isolates carrying *oxa58*, inspection of the assembly graphs in Bandage confirmed that all *oxa58* genes were flanked by IS*Aba3*. In most cases there was a single insertion site for IS*Aba3*. The surrounding sequence was extracted and annotated using the automated annotation pipeline Prokka version 1.10 (Seemann, 2014), followed by manual annotation of acquired resistance genes (by comparison with the arg-annot database; Gupta *et al.*, 2014) and insertion sequences (by comparison with the ISfinder database; Siguier *et al.*, 2006). The genetic contexts of *oxa40* and *ndm1* were investigated using the same approach.

### Analysis of the capsule locus in GC2

We used blastn version 2.2.31+ (Altschul *et al.*, 1990) to search the assemblies of GC2 genomes for the capsule biosynthesis locus (KL) flanking genes *mviN* (GenBank accession number CP001182, locus tag AB57_0088) and *IldP* (GenBank accession number CP001182, locus tag AB57_0116). For the 12 genomes in which the KL was split across multiple contigs, we used Bandage version 0.7.0 (Wick *et al.*, 2015) to inspect the assembly graph and to extract the sequence for the contiguous path through the KL. For each of the KL types identified in more than one GC2 isolate (KL2, KL49 and KL58), intra-KL sequence alignments were inferred using mafft version 7.245 (Katoh & Standley, 2013). Intra-locus diversity was then assessed by counting the variable sites in each of the alignments. Synteny alignments and sequence comparisons between KL2 and KL58 sequences, which share extensive sequence similarity, were made using blastn and Mauve version 2.3.1 (Darling *et al.*, 2004), and visualized using genoPlotR version 1.1 (Guy *et al.*, 2010) and act (Carver *et al.*, 2005). Assessment of sharing and reassortment of KLs within GC2, or between GC2 and CC10, via horizontal transfer was made after mapping reads to the KL2 reference sequence using RedDog, extracting SNPs as described above for phylogenetic analysis and visualizing recombination as described above (File S2). KLs were annotated as described in Kenyon & Hall (2013).

### Ancestral state reconstruction of GC2 capsule types

To infer ancestral KL types of lineages in the GC2 tree, we performed multistate ancestral state reconstruction using BayesTraits version 2.0 (Pagel *et al.*, 2004). We used the multistate implementation in BayesTraits to reconstruct the evolution of discrete KL types (four states: KL2, KL32, KL49 or KL58) on the set of phylogenetic trees (i.e. 72 000 pooled post-burn-in trees from the beast analysis). We used the MCMC implementation with a reversible jump and an exponential prior with a mean of 10. The analysis was repeated eight times, continuing each run for 10 × 10^6^ iterations and sampling from the posterior every 1000 iterations. Run log-files were examined using r.

## Results and Discussion

### Population structure of *A. baumannii* in the ICU

**Fig. 1.**
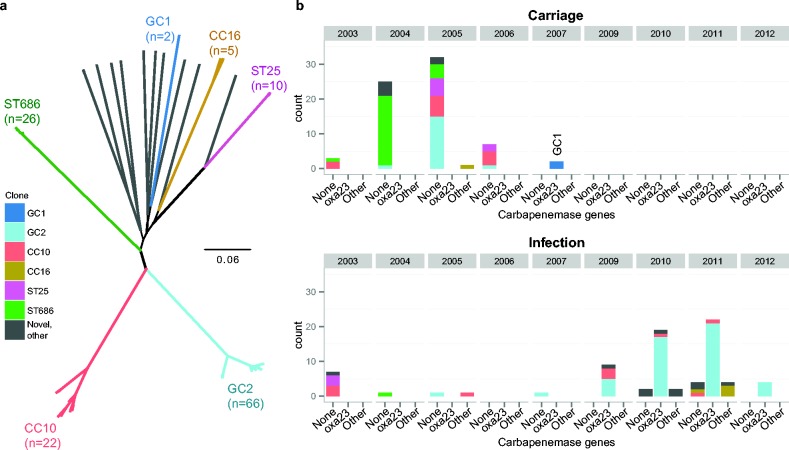
Phylogenetic and temporal structure of the ICU *A. baumannii* isolates. (a) Unrooted ML core genome phylogeny for all sequenced isolates. Clonal lineages are coloured and labelled according to their sequence type (defined using the Pasteur MLST scheme). (b) Temporal distribution of sequenced isolates according to presence of acquired *oxa23* or other carbapenemase genes; coloured by clone, as in (a). ‘Other’ includes carbapenemase genes *oxa40*, *oxa58* and *ndm1*.

The genomes of 147 *A. baumannii* isolates from VAP (*n* = 77) and asymptomatic carriage (*n* = 70) were available for analysis. MLST analysis of the genome sequences, using the seven-locus Institut Pasteur scheme, identified 21 sequence types (Table S1). Genome-wide SNP analysis identified 217 290 core genome SNPs, which were subjected to ML phylogenetic analysis. The core genome phylogeny and MLST structure identified similar population structures, and were used to classify the isolates into six strongly supported clonal groups plus an additional group of diverse strains (*n* = 16) (Fig. 1a). The six strongly supported clonal groups were: GC2 (ST2 and its single locus variants: ST570 and ST571), GC1 (ST1), CC10 (ST10 and its single-locus variants: ST23 and ST575), CC16 (ST16 and its single-locus variant: ST452), ST25 and ST686. The isolates previously typed as MLVA6 and MLVA8 (Nhu *et al.*, 2014) belonged to GC2 and CC10, respectively.

The temporal distribution of various genotypes is shown in Fig. 1(b). Of the sequenced isolates from 2003 to 2007 (*n* = 81), 86 % were from asymptomatic carriage. These early carriage isolates were genetically diverse, and included ≥ 10 isolates each from ST686 (32 %), GC2 (24 %), CC10 (20 %) and ST25 (12 %). The VAP isolates from this period (*n* = 11) fell within these same clonal groups, with the exception of a single VAP isolate of ST375, which was not represented within the asymptomatic carriage isolates. These data are consistent with the notion that carriage and VAP-associated isolates derive from the same population of *A. baumannii* on the ward, with opportunistic VAP infections arising following contact with a contaminated source (e.g. ventilator tubing). The later VAP isolates (*n* = 66), collected from 2009 to 2012, exhibited less genetic diversity and were dominated by GC2 (71 %), with smaller numbers of CC10 (9 %), CC16 (6 %) and occasional less commonly isolated strains (ST52, ST108, ST109, ST142 and three novel sequence types: ST754, ST755 and ST756).

**Fig. 2.**
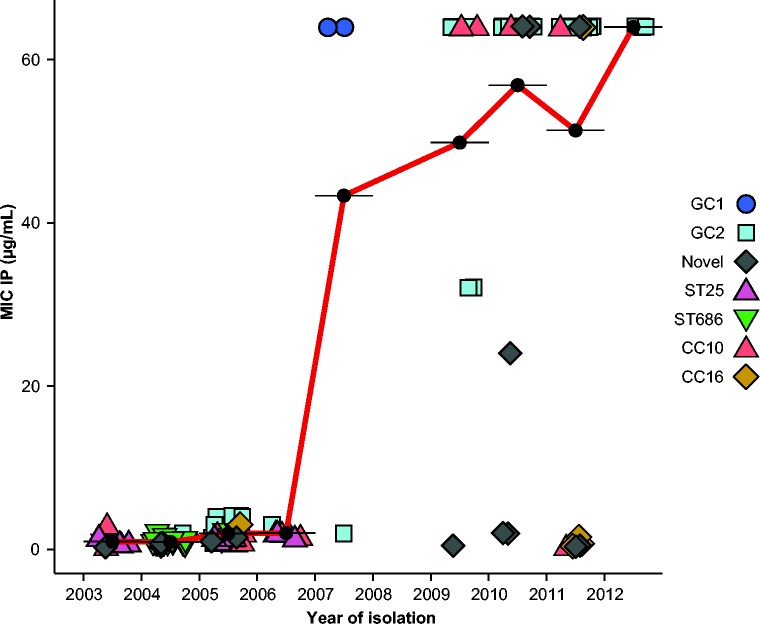
Temporal changes in the MICs of imipenem. Each point represents a single isolate, coloured to indicate clonal group. Black points indicate the mean MIC for each year, joined by red lines to show trend.

Most isolates (75 %) were resistant to amikacin, gentamicin, piperacillin/tazobactam, ciprofloxacin, ceftazidime and ceftriaxone, including isolates from all of the common clonal groups (GC1, GC2, CC10, CC16, ST109, ST25, ST52 and ST686; see Table S1). Fewer than 6 % of isolates were susceptible to two or more of these antibiotics (Table S1). Our previous review of imipenem-resistant VAP showed 15 % of VAP-associated *Acinetobacter* were resistant to imipenem during 2002–2007, rising to 86 % in 2010 (Nhu *et al.*, 2014). Eleven *A. baumannii* VAP isolates from 2002 to 2007 were available for sequencing in the present study and all of these were susceptible to imipenem (Fig. 2). Of the 70 carriage isolates from this period, all were imipenem-sensitive except for two GC1 isolates from 2007, which, notably, were the only GC1 isolates in this collection (MICs are shown in Fig. 2). Of the 66 post-2008 VAP isolates that were successfully sequenced, only nine (14 %) were susceptible to imipenem. The imipenem-resistant VAP isolates belonged to a variety of clones: GC2 (*n* = 47), CC10 (*n* = 5), CC16 (*n* = 1), and single isolates from ST52, ST108 and two novel singleton sequence types (ST755 and ST756) (Fig. 2). As GC2, CC10 and CC16 were also detected amongst the earlier isolates from the same ward, these data suggest that imipenem-resistant *A. baumannii* emerged on the ward on multiple occasions, within clones that had been previously circulating on the ICU.

### Genetic determinants of imipenem resistance

We identified at least one carbapenemase gene (*oxa23*, *oxa40*, *oxa58* or *ndm1*) in each imipenem-resistant isolate (Table S1). These were present in a variety of different genetic contexts and in variable copy numbers (Figs S1 and S2, summarized in Table 1). The *oxa40*, *oxa58* and *ndm1* genes were rare and mostly plasmid associated (Table 1, Note S1). We were able to completely assemble two novel circular plasmids carrying carbapenemase genes (Fig. S3; p255n_1 containing *oxa58*, deposited under GenBank accession number KT852971, and pBAL_204 containing *oxa40*, deposited under GenBank accession number KT946773).

**Table 1.** The genetic environment of acquired carbapenemase genes

Isolate or group	No. isolates	Imipenem MIC (μg ml^− 1^)	Gene	Mobile element	Depth ratio*	Chromosomal insertions of carbapenemases		Plasmid location§
						Copies†	Predicted site(s)‡	
GC2 (A)	1	64	*oxa23*	Tn*2006*	0.7	1	ABK1_1281 or ABK1_2910	–
GC2 (B)	2	32	*oxa23*	Tn*2008VAR*	0.7–0.9	1	ABK1_0148	–
	2	64	*oxa23*	Tn*2008VAR*	1.5–1.7	2	ABK1_0148+ABK1_3166	–
GC2 (C)	9	64	*oxa23*	Tn*2006*	1.5–1.8	2	ABK1_3492	–
	7	64	*oxa23*	Tn*2006*	2.4–2.7	3	ABK1_3492	
GC2 (D)	7	64	*oxa23*	Tn*2006*	0.8–0.9	1	ABK1_0857 or ABK1_2018 or ABK1_2567	–
GC2 (E)	19	64	*oxa23*	Tn*2006*	0.6–0.9	1	ABK1_2948 or ABK1_2960	–
CC10 (KL8)	3	64	*oxa23*	Tn*2006*	0.9–1.1	1	ABK1_3165	–
	1	64	*oxa23*	Tn*2006*	1.9	2	ABK1_3165	–
BAL_329 (CC10)	1	64	*oxa23*	Tn*2006*	0.7	1	ABK1_125 or ABK1_2164 or ABK1_2755	–
GC1	2	64	*oxa23*	Tn*2006*	0.4–0.5	–	–	a
BAL_212 (ST52)	1	64	*oxa23*	Tn*2006*	1.09	–	–	a
BAL_103	1	0.5	*oxa23*	Tn*2006*	0.07	–	–	b
UV 1268 (CC10)	1	1.5	*oxa58*	IS*Aba3*	0.38	–	–	c
BAL_283, BAL_287 (CC16)	2	0.75–1.5	*oxa58*	IS*Aba3*	0.5–0.8	–	–	c
BAL_255, 255_n (CC16)	2	64	*oxa58*	IS*Aba3*	0.5–0.6	–	–	c
			*ndm1*	Tn*125*	0.4–0.7	1	ABK1_1257	–
BAL_205, BAL_322	2	64	*oxa58*	IS*Aba3*	0.5–0.8	–	–	d
			*ndm1*	Tn*125*	0.4–0.7	–		d
BAL_204	1	24	*oxa40*	–	2.7	–	–	e

* Ratio of mean read depths for the carbapenemase gene versus known chromosomal genes (see Fig. S2).

† Carbapenemase gene copy number estimated from the depth ratio (see distributions in Fig. S1).

‡ Possible insertion site(s) for the carbapenemase-carrying mobile element (details of IS*Aba1* sites are shown in Fig. S5).

§ Key: a, Aci6 type plasmid, IS*Aba1* site in AbaR4; b, possibly lost during culture cross-contamination (note low read depth); c, novel plasmid p255n_1 (deposited under GenBank accession number KT852971); d, possible novel plasmids; e, novel plasmid pBAL_204 (deposited under GenBank accession number KT946773).

**Fig. 3.**
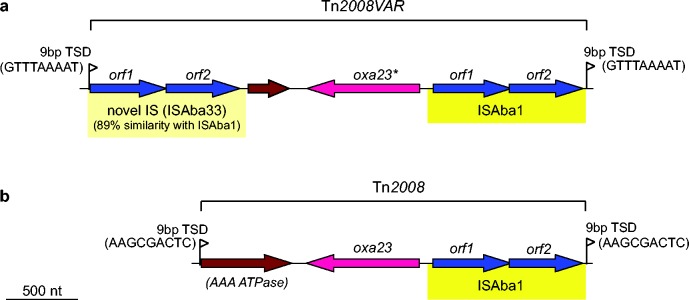
Genetic structure of the Tn*2008*-like transposon. Arrows indicate protein-coding genes, showing the direction of transcription and labelled with their assigned gene names. Genes are coloured to indicate function: blue, transposase; pink, carbapenemase; brown, other. Insertion sequences, which include transposase ORFs and flanking sequences, are shaded yellow. Flags indicate the presence of target site duplications (TSDs) that are formed upon transposition, which are labelled with the duplicated sequence. (a) Tn*2008*-like transposon of *A. baumannii* strain BAL_056 (Tn*2008VAR*, GenBank accession number KT852972, novel insertion sequence submitted to ISfinder as IS*Aba33*, the asterisk indicates a SNP leading to amino acid substitution L125V). (b) Tn*2008* transposon of *A. baumannii* strain 08325850 (GenBank accession number KP780408).

The most common carbapenemase gene was *oxa23*, which was present in 93 % of imipenem-resistant isolates, including 47 GC2 isolates, five CC10 isolates and the two GC1 carriage isolates from 2007 (Fig. 1b). The *oxa23* gene was generally encoded within a Tn*2006* transposon, present in one to three copies. Four GC2 isolates carried an *oxa23* variant (with a SNP leading to an amino acid substitution L125V) located within a Tn*2008*-like transposon (submitted as Tn*2008VAR* under GenBank accession number KT852972) that was flanked by 9 bp target site duplications, consistent with transposition via IS*Aba1* and a novel insertion sequence deposited under ISfinder as IS*Aba33* (Fig. 3). As the *oxa23* transposons were mobilized by insertion sequences, which appear in multiple copies in the GC2 genomes, it was not possible to determine the precise insertion sites of the *oxa23* transposons in each genome. For most *oxa23* isolates, the gene was present in one, two or three copies (Fig. S1) and the assembly data indicated that all the IS*Aba1* insertions were chromosomal (Table 1). In contrast, the two GC1 isolates had low copy number for *oxa23* (0.4–0.5) and harboured an Aci6-type plasmid carrying AbaR4 within which a copy of IS*Aba1* was present. This is consistent with plasmid carriage of the *oxa23* genes in these isolates, similar to an earlier report of GC1 isolates carrying Tn*2006* within AbaR4 in an Aci6 plasmid (Hamidian *et al.*, 2014).

### Emergence of imipenem resistance in *A. baumannii* GC2

The genomic data showed that the majority of imipenem-resistant VAP isolates belonged to GC2 (82.5 %) and all such strains carried the *oxa23* gene, consistent with our previous work linking imipenem resistance to an *oxa23*-positive clonal group with VNTR pattern MLVA6 (Nhu *et al.*, 2014). This pattern could result equally from the introduction of a new resistant GC2 subclone or from *de novo* emergence of resistance within the previously circulating GC2 population. We aimed to use the whole-genome data to distinguish between these possibilities and also sought to investigate whether the acquisition of *oxa23* occurred only once or on multiple occasions.

The genome-wide ML tree for the GC2 isolates showed that the imipenem-resistant VAP isolates fell into multiple groups, many of which were genetically distant from the earlier asymptomatic carriage isolates (File S1). However, it was clear from the distribution of SNPs in the alignment that there was substantial recombination affecting the GC2 genomes (Fig. S4a, File S1). As recombination can confound phylogenetic inference and intra-clonal relationships, we sought to recover a better estimate of vertical evolutionary signal within the GC2 isolates by first identifying and then removing recombinant SNPs prior to phylogenetic inference. Recombinant SNP detection was performed using both ClonalFrameML (Didelot & Wilson, 2015) and Gubbins (Croucher *et al.*, 2015). The Gubbins analysis yielded an apparently reliable recombination-filtered alignment of 457 SNPs, which displayed no spatial clustering of SNPs, a tree-like split network structure and strong clock-like (linear) temporal signal (*r*^*2*^ = 0.73, Fig. S4c; compare with the ClonalFrameML-filtered alignment in Fig. S4b or the raw data in Fig. S4a). Based on these estimates of 457 non-recombinant SNPs positions and comparing with the 13 493 recombinant SNPs, we estimate the ratio of SNPs introduced into the local GC2 population by recombination to those introduced by substitution mutation (*r*/*m*) to be ∼30. On the Gubbins recombination-filtered alignment, we performed a time-calibrated Bayesian analysis using beast, which yielded a substitution rate of 9.7 SNPs per year [95 % highest posterior density (HPD) 8–11, ESS >>1000].

**Fig. 4.**
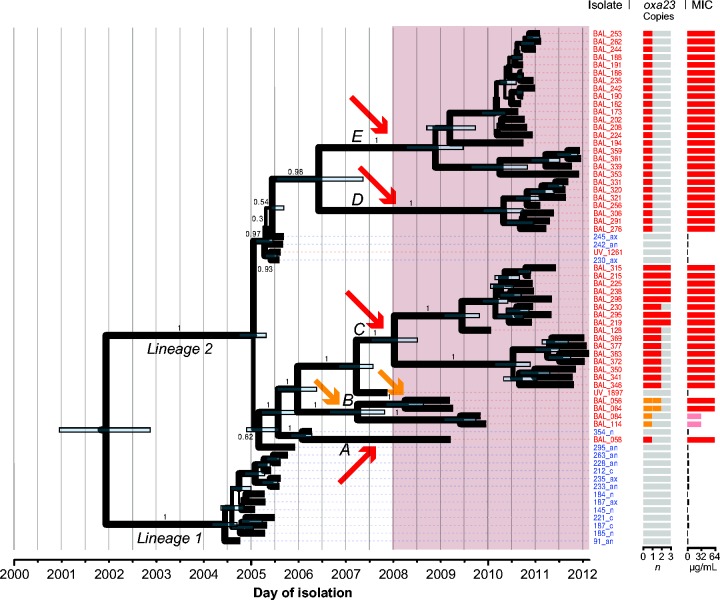
Recombination-filtered core genome phylogeny for GC2 *A. baumannii* in the ICU. beast maximum clade credibility tree; shading indicates the period during which imipenem was used for the empirical treatment of VAP in the ICU. Isolate labels are coloured to indicate source: red, VAP; blue, asymptomatic carriage. Node bars indicate 95 % HPDs for divergence dates; node labels and branch line thickness indicate posterior support. The two main lineages (1 and 2) and five imipenem-resistant subclades (A–E) referred to in the text are labelled, arrows indicate inferred *oxa23* carbapenemase acquisition events: red, Tn*2006*; orange, Tn*2008VAR*. *Oxa23* gene copy number and MICs for imipenem are indicated on the right.

In the resulting recombination-free GC2 beast tree (Fig. 4), the carbapenem-susceptible carriage isolates were divided into two well-supported lineages, which diverged around 2002 (95 % HPD 2001–2003, effective sample size >>1000). All of the imipenem-resistant GC2 VAP isolates belonged to lineage 2; however, these were paraphyletic and formed five distinct, strongly supported subclades (labelled A–E, Fig. 4). The most recent common ancestor (MRCA) of all imipenem-resistant GC2 VAP isolates was the same as the MRCA of the imipenem-susceptible lineage 2 carriage isolates, indicating that the resistant clades emerged from within the previously circulating susceptible population of lineage 2. The ML phylogeny showed a similar topology with 100 % bootstrap support for these subclades (Fig. S4c).

All imipenem-resistant GC2 isolates carried *oxa23* and each subclade had a unique clade-specific IS*Aba1* insertion site in the chromosome (Table 1, Fig. S5), consistent with unique insertions of Tn*2006* or the Tn*2008VAR* transposon in each subclade. The 95 % HPDs for the MRCA date of each subclade are consistent with these acquisition events occurring around or after the approval of imipenem for empirical treatment of VAP on this ICU, in 2008 (Fig. 4). Copy number of the *oxa23* gene was apparently associated with MIC: within subclade 2B (i.e. in the isolates containing the *oxa23* amino acid sequence variant in Tn*2008VAR*), isolates with one copy of *oxa23* displayed an MIC of 32 μg ml^− 1^ and isolates with two copies displayed an MIC of 64 μg ml^− 1^; outside of subclade 2B, isolates with at least a single copy of Tn*2006* displayed an MIC of 64 μg ml^− 1^. These data indicate that imipenem resistance arose *in situ* within the local GC2 population on at least five separate occasions, coinciding with the introduction of imipenem for empirical use on the ICU.

### Capsule replacement in GC2 *A. baumannii*

**Fig. 5.**
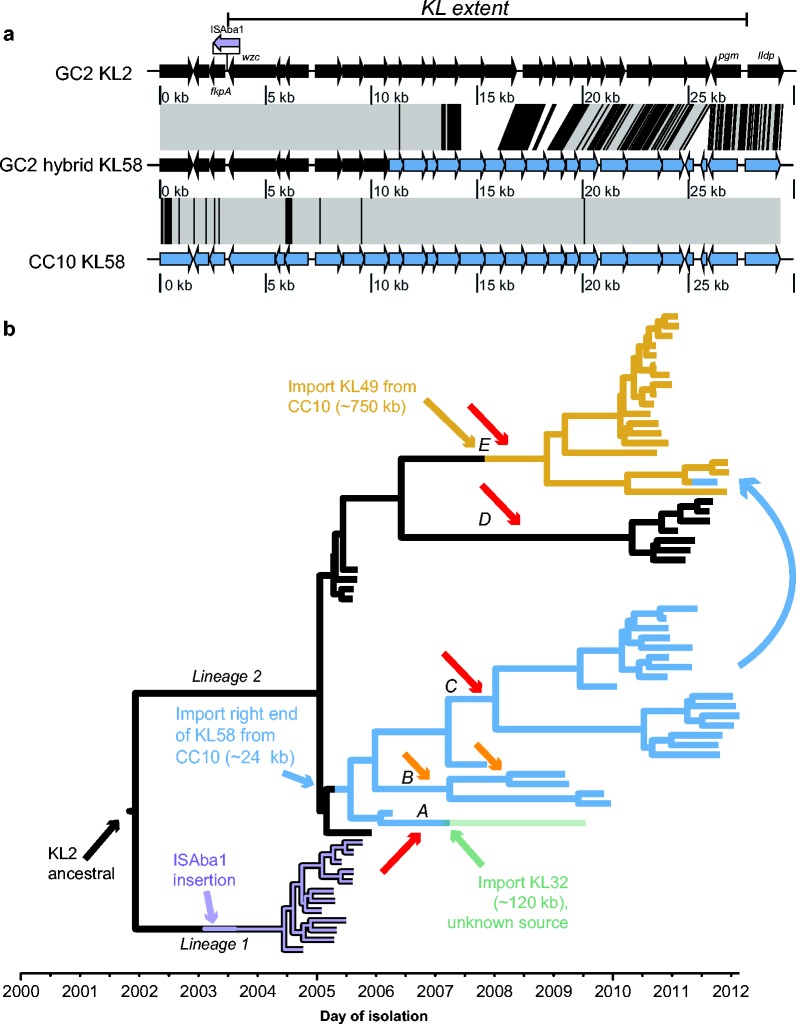
Capsule variation in the GC2 *A. baumannii*. (a) Schematic representation of a recombination event affecting the KL in GC2; the extent of the capsule biosynthesis gene cluster is indicated by the black bar. Arrows indicate protein-coding genes showing the direction of transcription, with selected genes labelled; KL2 genes are coloured black, typical KL58 genes are coloured blue; GC2 KL58 is a hybrid of KL2 genes (black) and KL58 genes (blue). Grey shaded blocks indicate sequence homology, black overlaid lines indicate SNPs. (b) Dated GC2 phylogeny showing KL recombination dynamics. Branches are coloured by capsule type and labelled by horizontal transfer events affecting the KL; subclades A–E are labelled with red and yellow arrows indicating *oxa23* transposon events (as in Fig. 4).

**Fig. 6.**
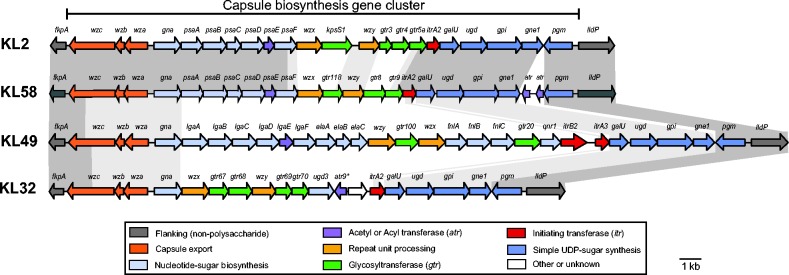
Genetic structure of KLs identified in the GC2 *A. baumannii* isolates. Gene clusters are drawn to scale against KL2 reference GenBank accession number KJ459911. KL32, KL49 and KL58 have been deposited under GenBank accession numbers KT359615, KT359616 and KT359617, respectively. KL names are indicated on the left. Arrows indicate protein-coding genes, showing the direction of transcription and labelled with their assigned gene names; an asterisk indicates an internal frameshift. Genes are coloured by the predicted functional group of their gene product according to the legend. Grey shaded blocks indicate sequence homology.

The regions we identified as being affected by recombination in the local GC2 population included the KL, which encodes capsule biosynthesis. Vaccines that target capsule polysaccharides are an alternative to antibiotics in the control of pan-resistant Gram-negative bacterial infections. Notably, we detected four distinct KLs amongst the GC2 isolates, consistent with capsule replacement induced via homologous recombination. Amongst the 66 GC2 isolates, 24 carried the KL2 gene cluster (Kenyon & Hall, 2013), 18 carried KL49 (Kenyon *et al.*, 2015), 23 carried a novel gene cluster that we designated KL58 (Figs 5a and 6) and one carried KL32 (same sequence as PSgc21 in Hu *et al.*, 2013). Variation in KLs was mirrored by allelic variation in the *gpi* locus, which is utilized in the University of Oxford MLST scheme (Table S1) and is located within the KL. The genetic structures of these four KLs (Fig. 6) revealed distinct saccharide processing genes in each locus; hence, they are expected to produce distinct surface polysaccharides, although to date the precise structures have been determined only for K2 and K49 (Kenyon *et al.*, 2014; Senchenkova *et al.*, 2014; Vinogradov *et al.*, 2014).

Nearly all of the early GC2 isolates carried KL2, which was not represented in the non-GC2 isolates. Treating KL type as a discrete trait, ancestral state reconstruction on the GC2 beast tree set identified KL2 as the most likely type in the MRCA of all GC2 genomes in this collection (Fig. S6). A maximum of four pairwise SNPs were detected amongst the KL2 sequences and the SNPs flanking the KL2 locus did not vary markedly between our GC2 KL2 isolates, consistent with shared inheritance of the KL2 region from a common ancestor carrying this locus and flanking sequence. A *de novo* assembly and alignment of all KL2 sequences identified an insertion of the IS*Aba1* transposase next to *wzc*, the first gene of KL2, in the GC2 lineage 1 isolates; however, this is unlikely to affect expression of KL genes as it is located downstream of the *wzc* ORF (Fig. 5a).

The majority of the remaining non-KL2 GC2 isolates harboured either the KL49 or KL58 capsule genes (Fig. 5b). Notably, both KL49 and KL58 were also identified in CC10 carriage isolates and VAP isolates from 2003 to 2006, providing a potential source for these sequences within the local *A. baumannii* population. Detailed inspection of the KL and surrounding sequences in GC2 and CC10 isolates indicated that the KL49 GC2 strains most likely resulted from a single importation of 750 kb (spanning the KL49 locus) into the ancestor of GC2 clade 2E, derived from co-circulating CC10 KL49 strains (or from a third donor that also donated KL49 and flanking sequence to the co-circulating GC2 and CC10 clones) (Fig. 5b). Two variants of KL58 were identified: one in GC2 and the other in CC10. The GC2 KL58 sequence appears to be a hybrid of KL2 and the CC10 KL58 sequence (Fig. 5a), resulting from a local homologous recombination event, in which 24 kbp towards the 3′ of the KL58 locus was imported from CC10 (or from a third donor that also donated KL58 and flanking sequence to the co-circulating GC2 and CC10 clones). The GC2 KL58 isolates formed a single monophyletic group (subclades 2A–C, MRCA around 2005; Fig. 5b) consistent with a single import resulting in the formation of the recombinant KL58 locus. However, within this group was a single isolate (BAL_058) that carried a distinct KL, KL32, which was not identified in any other isolate (Fig. 5b). SNP variation on either side of the KL32 locus was indicative of a recombination import spanning ∼120 kb. In addition, a single isolate (BAL_339) nested within the KL49 subclade 2E carried a KL58 sequence identical to the hybrid KL58 sequence found in GC2 2A–C isolates, suggesting it was acquired from a GC2 strain.

Capsule replacement is not well studied amongst Gram-negative bacteria, but has recently been observed in multiply antibiotic-resistant epidemic clones of *A. baumannii* (Kenyon & Hall, 2013; Kenyon *et al.*, 2014, 2015), *K. pneumoniae* CC258 (Wyres *et al.*, 2015) and *E. coli* ST131 (Alqasim *et al.*, 2014). Potential drivers of capsule diversification include host immunity, phage predation and also antimicrobial resistance as it has recently been shown that capsule variation in *A. baumannii* is associated with variation in susceptibility to multiple antimicrobials (Geisinger & Isberg, 2015). Here, two of the KL replacement events resulted in novel clades, i.e. KL58 GC2 (2A–C) and KL49 GC2 (2E), that were successful in persisting within the local population for several years. The local maintenance of KL2 GC2 alongside these newer variants indicates that if there was selection for capsule variation in the local population, this was not strong enough to drive clonal replacement of KL2 GC2 in this instance.

## Conclusions

Our analyses indicate that the recent surge of imipenem-resistant VAP in this ICU was attributable to multiple imipenem-resistant clones, the majority of which arose from within the local *A. baumannii* population via the acquisition of mobile carbapenemase genes. Whilst precise details of imipenem usage are not available, these acquisition events occurred at around the same time at which imipenem was approved for empirical treatment of VAP on the ward, consistent with the hypothesis that the change in treatment strategy drove rapid changes in the local population of *A. baumannii* and resulted in the repeated emergence of multiple imipenem-resistant subclones from within the local population. This scenario contrasts with recent genomic investigations of epidemics of carbapenem-resistant Gram-negative clones in other hospital settings, which have reported transmission of a singular resistant clone (Mathers *et al.*, 2015; Snitkin *et al.*, 2012), competition between resident and incoming resistant clones (Brañas *et al.*, 2015) or transmission of a resistant plasmid amongst diverse bacterial hosts (Mathers *et al.*, 2011, 2015).

Diversification of capsule types within GC2 appears to have commenced at around the same time as the emergence of imipenem resistance in the population and was driven by homologous recombination. The fact that we were able to identify likely donors for two of the imported capsule loci amongst previously circulating strains of the second most common *A. baumannii* clone on the ward (CC10) is notable. It suggests that whilst the population of *A. baumannii* on the ward is diverse, consisting of multiple strains, this population may nevertheless have remained ‘closed’, with evidence of reassortment between strains, but limited evidence for the introduction of new genetic material – a finding consistent with that observed in *Enterococcus faecium* by van Hal *et al.* (2016). Interestingly, the earliest examples of the *oxa23* carbapenemase on the ward were two GC1 carriage isolates from 2007, which are the only examples of GC1 we identified in this setting and provide a potential external source for the introduction of the *oxa23* carbapenemase into the local *A. baumannii* population.

Similar to the findings of Wright *et al.* (2014), our investigations underscore the importance of whole-genome sequence analysis to tease apart fine-scale evolutionary events within bacterial pathogen populations, as our earlier MLVA and PCR analyses could not distinguish the presence of distinct subclades of GC2 that had independently evolved imipenem resistance.
